# Creating a next-generation phenotype library: the health data research UK Phenotype Library

**DOI:** 10.1093/jamiaopen/ooae049

**Published:** 2024-06-17

**Authors:** Daniel S Thayer, Shahzad Mumtaz, Muhammad A Elmessary, Ieuan Scanlon, Artur Zinnurov, Alex-Ioan Coldea, Jack Scanlon, Martin Chapman, Vasa Curcin, Ann John, Marcos DelPozo-Banos, Hannah Davies, Andreas Karwath, Georgios V Gkoutos, Natalie K Fitzpatrick, Jennifer K Quint, Susheel Varma, Chris Milner, Carla Oliveira, Helen Parkinson, Spiros Denaxas, Harry Hemingway, Emily Jefferson

**Affiliations:** SAIL Databank, Medical School, Swansea University, Swansea, SA2 8PP, United Kingdom; Health Informatics Centre, School of Medicine, University of Dundee, Dundee, DD1 9SY, United Kingdom; School of Natural and Computing Sciences, University of Aberdeen, Aberdeen, AB24 3UE, United Kingdom; SAIL Databank, Medical School, Swansea University, Swansea, SA2 8PP, United Kingdom; SAIL Databank, Medical School, Swansea University, Swansea, SA2 8PP, United Kingdom; SAIL Databank, Medical School, Swansea University, Swansea, SA2 8PP, United Kingdom; SAIL Databank, Medical School, Swansea University, Swansea, SA2 8PP, United Kingdom; SAIL Databank, Medical School, Swansea University, Swansea, SA2 8PP, United Kingdom; Department of Population Health Sciences, King’s College London, London, SE1 1UL, United Kingdom; Department of Population Health Sciences, King’s College London, London, SE1 1UL, United Kingdom; Adolescent Mental Health Data Platform and DATAMIND, Swansea University, Swansea, SA2 8PP, United Kingdom; Adolescent Mental Health Data Platform and DATAMIND, Swansea University, Swansea, SA2 8PP, United Kingdom; SAIL Databank, Medical School, Swansea University, Swansea, SA2 8PP, United Kingdom; Institute of Cancer and Genomic Sciences, University of Birmingham, Birmingham, B15 2TT, United Kingdom; Institute of Cancer and Genomic Sciences, University of Birmingham, Birmingham, B15 2TT, United Kingdom; Institute of Health Informatics, University College London, London, NW1 2DA, United Kingdom; School of Public Health and National Heart and Lung Institute, Imperial College London, London, W12 0BZ, United Kingdom; Health Data Research United Kingdom, London, NW1 2BE, United Kingdom; Health Data Research United Kingdom, London, NW1 2BE, United Kingdom; European Molecular Biology Laboratory, European Bioinformatics Institute (EMBL-EBI), Welcome Trust Genome Campus, Hinxton, Cambridge, CB10 1SD, United Kingdom; European Molecular Biology Laboratory, European Bioinformatics Institute (EMBL-EBI), Welcome Trust Genome Campus, Hinxton, Cambridge, CB10 1SD, United Kingdom; Institute of Health Informatics, University College London, London, NW1 2DA, United Kingdom; University College London Hospitals National Institute of Health Research Biomedical Research Centre, London, NW1 2BU, United Kingdom; British Heart Foundation Data Science Center, Health Data Research United Kingdom, London, NW1 2BE, United Kingdom; Institute of Health Informatics, University College London, London, NW1 2DA, United Kingdom; University College London Hospitals National Institute of Health Research Biomedical Research Centre, London, NW1 2BU, United Kingdom; Health Informatics Centre, School of Medicine, University of Dundee, Dundee, DD1 9SY, United Kingdom; Health Data Research United Kingdom, London, NW1 2BE, United Kingdom

**Keywords:** electronic health records, phenotyping, public health informatics, algorithms, application programming interface, medical informatics

## Abstract

**Objective:**

To enable reproducible research at scale by creating a platform that enables health data users to find, access, curate, and re-use electronic health record phenotyping algorithms.

**Materials and Methods:**

We undertook a structured approach to identifying requirements for a phenotype algorithm platform by engaging with key stakeholders. User experience analysis was used to inform the design, which we implemented as a web application featuring a novel metadata standard for defining phenotyping algorithms, access via Application Programming Interface (API), support for computable data flows, and version control. The application has creation and editing functionality, enabling researchers to submit phenotypes directly.

**Results:**

We created and launched the Phenotype Library in October 2021. The platform currently hosts 1049 phenotype definitions defined against 40 health data sources and >200K terms across 16 medical ontologies. We present several case studies demonstrating its utility for supporting and enabling research: the library hosts curated phenotype collections for the BREATHE respiratory health research hub and the Adolescent Mental Health Data Platform, and it is supporting the development of an informatics tool to generate clinical evidence for clinical guideline development groups.

**Discussion:**

This platform makes an impact by being open to all health data users and accepting all appropriate content, as well as implementing key features that have not been widely available, including managing structured metadata, access via an API, and support for computable phenotypes.

**Conclusions:**

We have created the first openly available, programmatically accessible resource enabling the global health research community to store and manage phenotyping algorithms. Removing barriers to describing, sharing, and computing phenotypes will help unleash the potential benefit of health data for patients and the public.

## Background and significance

### Electronic phenotyping

Electronic health records (EHRs) and other routinely collected data sources contain a wealth of information that can be used for the benefit of patients and populations.^1^ Secondary analysis of such data has become increasingly important in recent years, a trend which the COVID-19 pandemic accelerated.^2^^,^^3^

One of the primary challenges in this field is mapping data (which is often messy and has limitations related to its operational purpose) to the routine patient characteristics and events that are of interest for analysis. For example, a researcher may want to identify patients diagnosed with depression,^4^ people at high risk of severe COVID-19,^5^ or prescriptions of anti-diabetic medication.^6^ It is rarely straightforward to do so within datasets used for analysis, which may include sources such as coded primary care records, insurance payment information, or free text notes.^7^ To address these challenges, researchers apply a variety of techniques, including curation of clinical code lists, rule-based algorithms, machine learning techniques, natural language processing, etc.^8–11^

This work is increasingly recognized as a distinct discipline within health data analysis, often referred to as electronic phenotyping.^12^^,^^13^ The definitions that are created (or phenotyping algorithms) are not mere technical minutiae but important first-order outputs of health data research. An analysis is only as good as the underlying definitions it is based on. Furthermore, phenotypes are useful building blocks that can often be reused in new contexts, increasing both the efficiency and rigor of research—for example, a definition in a research study on smoking might be useful to hundreds of other researchers who want to include smoking status as a lifestyle risk factor in different analyses.^14–16^ As highlighted in the CODE-EHR reporting framework for health data research, it is essential that published research “fully detail how conditions and outcome events were defined, allowing other researchers to identify errors and repeat the process in other datasets.”^17^

### Sharing phenotypes

Existing mechanisms of sharing phenotype definitions when publishing a paper include putting clinical code lists in Supplementary material or sharing scripts on GitHub.^18^^,^^19^ However, both have major limitations. For example, the phenotypes are not discoverable except by reading the paper in detail, limiting the potential for beneficial reuse of methods; and without any standardization of format or information collected, it is unrealistic to expect high-quality, complete definitions that can readily be adopted by others.

There is a widely recognized need for resources specifically to support sharing phenotypes, and several ongoing projects to create libraries or repositories of phenotypes.^20–31^ In a recent paper, we proposed requirements for phenotype libraries, focusing on 5 key areas^32^: (1) authoring/modeling, (2) refining/logging, (3) implementation, (4) validating, and (5) sharing. In reviewing existing libraries^21–27^ against these requirements, we identified that—while much excellent work is being done—there are gaps in the state of the art both in terms of functionality and scope. In this manuscript, we present a next-generation phenotype library developed ensuring design based on the proposed desiderata,^32^ to promote open-science and a more interoperable phenomics knowledge base.

### Desiderata for next-generation FAIR phenotype libraries

The FAIR (Findability, Accessibility, Interoperability, and Reusability) principles,^33^ published in 2016, have become important guiding principles within the health data science community. In our previous work,^32^ we examined existing phenotype tooling and libraries to identify best practices for authoring and storing FAIR phenotype definitions and translated these into a set of desiderata for the development of future libraries.

Among our findings, we concluded that supporting the authoring (and subsequent storage) of a range of different definition types under a pre-defined model improves intelligibility and thus reusability. Formally recording the provenance of a phenotype definition and the relationship between definitions has similar effects. Furthermore, improving the portability of definitions—by building libraries that support the generation of computable phenotypes, which can be executed against multiple data standards, from their definition counterparts—contributes to interoperability. Finally, we found that offering a standard API improves accessibility, while enhanced search features and extensive metadata (eg, validation information) can increase findability.

### Existing libraries landscape

Several resources have been created with the intent of documenting phenotype definitions, such as CALIBER (UK),^21^^,^^22^ ClinicalCodes (UK),^20^^,^^23^ Opencodelists (UK),^24^ PheKB (USA),^25^ the Manitoba Centre for Health Policy (MCHP) concept dictionary and glossary (CANADA),^26^ and OHDSI Phenotype Library (EUROPE).^27^^,^^28^ A US based initiative by the Department of Veterans Affairs Office of Research and Development for the development of Centralized Interactive Phenomics Resource (CIPHER) is also in progress though not publicly accessible yet.^34^ Additionally, research groups/projects across the UK (eg, Child Health Informatics Group at University College London,^35^ a research project at London School of Hygiene & Tropical Medicine,^29^ a research project on severe mental illness by the University of Bristol,^30^ and Cambridge Research Methods Hub at University of Cambridge^31^) also created independent web-based resources to share their code lists.

These existing resources all aim to meet the needs described above in some way, and implement important features such as search, upload, and re-use capabilities.^36^ However, a qualitative study involving multi-disciplinary participants focusing on their experience with existing phenotype libraries and their future requirements and recommendations concluded that there is a need to enable ease-of-use, improve documentation, and make definitions more transparent.^37^ The authors also concluded that there is no single general-purpose library with broad support for the variety of data settings and use cases in the health data analysis field. More details on the features of some existing libraries with comparison to the library presented in this manuscript are demonstrated in Table 3.

**Table 1.** Description of webpages of the HDR UK Phenotype Library.

Menu option	Description
Home	Home menu displays descriptive statistics of available contents: number of phenotypes, concepts, clinical codes, data sources, and coding systems. The page also has a full text search box, key definitions (eg, “what is a phenotyping algorithm,” “key principles,” “how are we driving this work,” etc), and a link to add a phenotype page.
Phenotypes	Phenotype’s menu lists all publicly available phenotypes. It allows full-text search as well as offering filtering options: Type of phenotype (disease or syndrome, biomarker, lifestyle risk factor, etc) Collection (CALIBER, ClinicalCodes, BHF Data Science Centre, etc) Coding system (Read codes v2, SNOMED CT codes, ICD-10 codes, etc) Data Source (CPRD Gold, CPRD Aurum, Hospital Episode Statistics Admitted Patient Care, etc) Search Interval (last week, last month, last 6 months, etc) Authorship Once a phenotype is selected from the list, a phenotype page presents: Phenotype Name (with list of authors) Metadata Type of phenotype—text field—eg, disease or syndrome, biomarker, etc ID—text field—eg, PH123 Version ID—numeric field—eg, 4, 5, 6 Data sources—text field—eg, CPRD GOLD, CPRD AURUM Valid event data range—text field—eg, 01/01/2010–30/06/2020 Sex—text field—eg, male or female Coding system—text field—eg, Read code v2, SNOMED CT Collection—text field—eg, BREATHE, CALIBER, ClinicalCodes repository Human readable definition Implementation details (if available—either in descriptive form or graph or computable form) Publication Clinical code list(s) API endpoints to access phenotype details and clinical code lists in CSV/JSON/XML formats Version history
Concepts	The concepts page lists all publicly available concepts. A concept is a single list of codes relating to one data source/coding system (eg, primary care or secondary care/Read code version 2 or ICD-10 code). A phenotype algorithm may consist of one or more concepts. For example, a phenotype of a heart failure (phenotype ID: PH182) is made up of 3 concepts: C1206 (concept ID) represents Read code v2 codes, C1207 (concept ID) represents CPRD med codes from primary care data, and C1208 (concept ID) represents ICD-10 codes from secondary care data.
API	API documentation page listing all the API endpoints.
About	This menu has the following sub-menu options: About the project: this page has details about the HDR UK phenotype library covering challenges, objectives, key principles, future plans, etc. Team: brief biography of team members. Technical details: this page has details about the phenotype library inclusion criteria and details about the YAML template to add a phenotype. COVID-19 response: a page that explains the COVID-19 initiative for open-access COVID-19 related phenotypes. Publications: list of published articles by the team related to phenotypes Collections: this sub-menu has landing pages with brief description of each type of collections such as BREATHE, BHF Data Science Centre, etc. Reference data: a list of data sources, coding systems, collections, phenotype types, etc, and will be used for referencing these sources when preparing YAML format for adding/submitting a phenotype to the library. Contact us: the page allows users to request user account, reporting issues and features, and general queries.
Log in	Access to phenotypes does not require a user registration; however, to add phenotypes a user registration is required which can be requested through contact us page (available as sub-menu under *About* page).

**Table 2.** Phenotype categories and counts.

Phenotype categories	*N* (%)
Disease or syndrome	837 (79.79)
Drug	142 (13.54)
Biomarker	38 (3.62)
Lifestyle risk factors	31 (2.96)
Surgical procedure	1 (0.09)
**Total**	**1049 (100)**

**Table 3.** Comparison of the HDR UK Phenotype Library with other similar Phenotype Libraries regarding the desiderata we proposed recently for developing the next-generation of phenotype libraries.

Sr. No.	Principle	HDR UK Phenotype Library	**PheKB** ^25^	**Opencodelist** ^24^	**CIPHER** ^34^	**ClinicalCodes** ^20^	**MCHP concept dictionary and glossary** ^26^
1	Support modeling languages	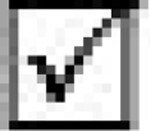	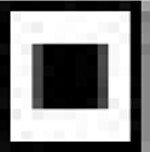				
2	Support NLP-based and ML-based definitions						
3	Support multi-dimensional descriptions	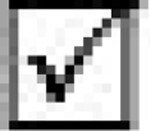					
4	Support versioning and data provenance	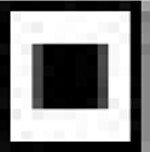		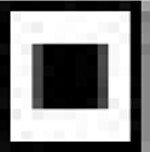			
5	Support modular relationships between phenotypes	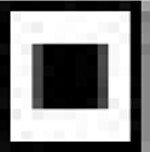					
6	Communicate implementation information in the model	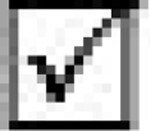		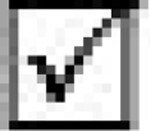			
7	Support tooling that provides multiple programming language implementation	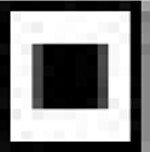					
8	Support tooling that provides connectivity with multiple data standards						
9	Support a defined validation process	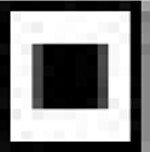	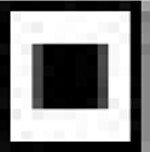				
10	Automate multiple validation techniques						
11	Enable feedback	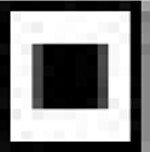	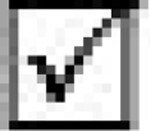	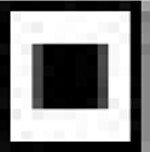		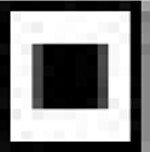	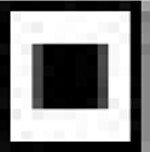
12	Expose a standard API	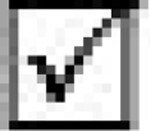					
13	Offer advanced search capabilities	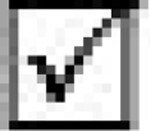	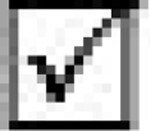	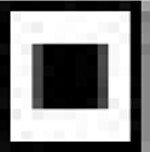	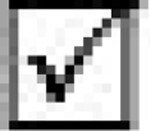	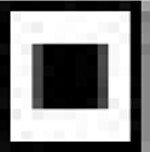	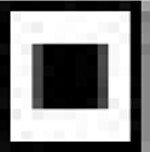
14	Include comprehensive metadata	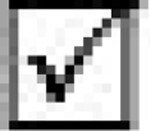	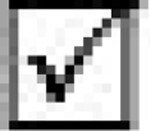	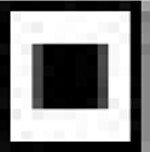	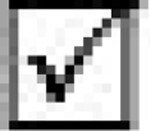	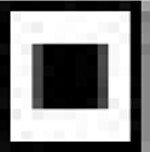	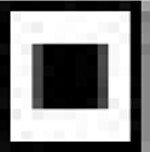

The symbol 
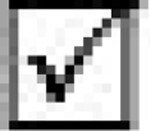
 indicates yes, the symbol 
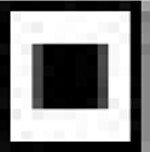
 indicates somehow/partial and the symbol 

 indicates no.

### Objectives and justification

Given the increasing importance of routinely collected health data analysis and the rise of the discipline of electronic phenotyping, there is clear benefit to ensuring that data users have effective tools to manage phenotypes. Our previous work sets out a case for what features are important in such a tool, and a survey of the state-of-the-art has shown that there is no extant solution that meets all those needs—at least not among open tools that are accessible to all users.

Therefore, we set out to create a new phenotype library for creating, managing, and sharing phenotype definitions. This tool aims not only to meet the needs of researchers connected to our funder, Health Data Research UK (HDR UK), but also to serve as a global resource for health data users in a wide variety of contexts.

We expect this tool to support improvements in the quality of health data analysis through greater transparency and reproducibility as well as increased efficiency by reducing barriers to sharing and reuse. Together, these improvements serve the larger goal of maximizing the benefit of health data sources to population health and wellbeing.

## Methods

### Prior work

We brought 2 existing phenotype library resources to this work that served as a starting point for our development of this project:

#### CALIBER

CALIBER was established in 2012 providing a common analytics approach across 4 national linked datasets (Clinical Practice Research Datalink [CPRD],^38^ Hospital Episode Statistics [HES],^39^ Office for National Statistics [ONS],^40^ and the Myocardial Ischaemia National Audit Project [MINAP]^41^).^21^^,^^22^ While the initial focus was cardiometabolic research, the resource expanded and enabled research across diseases at scale. Early on, CALIBER established a robust EHR phenotype creation and validation approach, which was applied in all phenotyping algorithms that were created as part of the project. These were deposited in an open access resource, the CALIBER Portal, which formed one of the basis of the Phenotype Library.

#### Concept Library

The Concept Library is a web application developed to enable researchers working in the Secure Anonymised Information Linkage (SAIL) Databank to create, document, and share phenotypes based on clinical code lists.^42^ The Phenotype Library has been built on the Concept Library codebase, as a shared development project with the Concept Library development team.

### Prototype

As an initial step, we developed a prototype version of the library using a simple Jekyll/Netlify^43^^,^^44^ site generator where R Markdown files were rendered as static HTML pages. This pilot project served several goals. First, we hoped to realize immediate benefit by rapidly delivering a useful minimum viable product. Second, the pilot enabled collection of phenotype content while the main library was being delivered: researchers were invited to contribute phenotypes via a Google Form, which were then stored in GitHub in a format that could be easily loaded into the production library later. Third, the prototype helped us define requirements for the final library through real-world use as well as serving as an initial design example that potential users could review and critique.

### Users and interface

Phenotypes are potentially relevant to a wide variety of health data users, including researchers, clinicians, healthcare analysts, etc. For pragmatic reasons of limiting scope and leveraging existing skills and relationships, the requirements of health data researchers were the primary focus of the first version of the phenotype library.

A user experience (UX) expert (C. Oliveira—a co-author in this manuscript) in the team organized interview sessions with users to understand their experience of using prototype library static page(s) so a better and improved version of the library could be designed and developed. The interviews were conducted based on the 5 act interview structure^45^: (1) friendly welcome, (2) context questions, (3) prototype introduction, (4) tasks to understand how user reacts to the prototype, and (5) debrief. Taking into account users’ feedback, the pages of the library were designed using Figma^46^ (a collaborative interface design tool). A description of each web page of the live phenotype library^47^ is presented in Table 1.

### Design principles

The design was guided by the proposed desiderata for next-generation phenotype libraries^32^:

1. **Authoring/modeling—**A standard model for phenotype metadata, with multiple levels of optional implementation information.
2. **Refining/logging—**Phenotype version control and edit history; modular phenotype relationships.
3. **Implementation—**Integration with phenoFLOW to support workflow-based implementations.
4. **Validating—**Ability to capture existing validation information.
5. **Sharing—**Searchable and browsable content via a web interface as well as accessibility via an API. Discoverability via dataset metadata.

#### Authoring/modeling

The phenotype model reflects the goal of “supporting multi-dimensional descriptions” articulated in our previous work: it should be possible to articulate a high-level, conceptual model that can be extended with implementation-specific detail. The library’s model for phenotype metadata aims to support the community of researchers and other health data users as broadly as possible. It includes a unique ID and version; information about the type of phenotype; dataset context and coding system (where applicable); and information about the definition, implementation, and validation of the phenotype (the full definition can be found in the Supplementary material S1).

This high-level model can be extended with more implementation-specific detail. Where a phenotype uses clinical codes, the definition can be extended with one or more sets of codes used to implement it; clinical codes and associated metadata are viewable and searchable via the web interface as well as accessible via specific API calls. Another level of implementation detail is enabled via the link to a phenoFLOW implementation (see the Supplementary material S2 for an example).

Aligned with the data model designed to support a broad user base, we have made the library available to users to directly contribute content: users can request a user account via the “Contact Us” page on the website. Phenotypes can be uploaded and edited by the community using an API-based process. A flexible permissions model gives the ability to control who can see content in development and lets users choose when to publish their content to make it publicly visible.

#### Refining/logging

The library keeps a complete history of changes to phenotypes; users are able to explore and use previous versions through the version history on the website and via the API. Each phenotype has a URL that directs to its latest version as well as permanent URLs that point to each previous version. The latter enables citing the exact version of a phenotype definition used in a paper, etc.

In the desiderata paper, we emphasized the importance of being able to define modular relationships between phenotypes—such as using existing phenotypes as building blocks to construct new ones. The library enables a phenotype to include one or more other existing phenotypes in its definition.

#### Implementation

Supporting computability was highlighted as a requirement in our previous work and is reflected in the library’s design. Making all content accessible programmatically via an application programming interface (API) is a key enabler of computability, and this was a core requirement of the library.

The API follows the design pattern of the representative state transfer (REST) architecture.^48^ Calls have been implemented for all library functionality, including search, accessing individual phenotype metadata, accessing clinical code lists, creating and editing phenotypes, etc. The API is versioned to enable future updates and changes, while maintaining backwards compatibility. API documentation is available on the HDR UK Phenotype Library.^49^

API clients can enable the API to be used more easily within a particular programming language or setting. One example client is the ConceptLibraryClient R package,^50^ allowing R users to easily integrate phenotypes into their data analysis, as well as upload content from R with a template-based system. The client was implemented in R as a pragmatic choice to support a particular active user base; other similar work may be undertaken in other languages in the future.

One barrier to the wide use of computable phenotypes is the challenge of bringing the phenotypes to the data. Health data research often takes place within trusted research environments (TREs)—secure computing environments that do not have access to the internet.^51^ We created a security model that enables phenotypes to be accessed (but not edited or created) within a TRE, using a separate version of the library application that runs with reduced permissions. This model is currently operational to make the library available within the SAIL Gateway, a TRE that holds health data for researchers in Wales.^52^

Besides these general features supporting computability, a specific fully computable phenotype model is supported via integration with the Phenoflow library.^53^ Phenoflow uses the workflow paradigm, connecting a high-level description of the steps required to derive a phenotypic cohort to one or more computable forms both at the modeling and implementation (eg, via the Common Workflow Language [CWL]) levels.^54^

#### Validation

The initial version of the library supports a minimum set of validation features. As articulated in our previous work, validation is critical to high-quality phenotypes. However, in terms of priority, we believe that broad adoption and enabling discoverability largely precede validation.

Mandating rigorous validation, or only accepting content that is considered a “gold standard”, may seem like laudable goals to improve the quality of research. However, in practice setting, the bar too high will result in much of the existing work remaining invisible and undiscoverable. We believe that instead accepting a much broader array of content will create a context and resource that can drive future validation and improvement.

The library currently implements 2 validation features:

- Users can include existing validation information with phenotypes, where available.
- There is a simple approval process for publishing phenotypes, which involves one of the Phenotype Library team reviewing the phenotype to ensure that it is appropriate content submitted in good faith and fully documented.

#### Sharing

The Phenotype Library web interface enables searching for phenotypes, including filtering on several categories. The API described above provides another mechanism for sharing content.

Further content visibility is achieved by integration with a public dataset metadata resource. The HDR UK Innovation Gateway is a tool that is designed to be a central clearinghouse for health dataset metadata in the UK, supporting dataset discovery and use for research. It has broad adoption, holding information on over 800 datasets.^55^ A bidirectional link between the Gateway and the Library is implemented using their respective APIs. Within a phenotype, users can click on a dataset and be taken to detailed metadata on that dataset if it exists in the Gateway. Likewise, the Gateway allows users to find phenotypes associated with a particular dataset, supporting discoverability.

## Results

The prototype library was launched in November 2020 with an initial collection of 308 phenotypes. Over the pilot phase of the project, this grew to 330 phenotypes via user submissions. This was followed by the official launch of the production Phenotype Library in October 2021. As of the submission of this manuscript, it holds 1049 phenotypes comprising a total of 2317 code lists and more than 200 000 clinical codes; 348 of these phenotypes link to computable implementations in PhenoFLOW.

Phenotypes are defined against 40 health data sources using sixteen different clinical coding terminologies, including SNOMED-CT,^56^ Read,^57^ ICD-10,^58^ and others.^59^^,^^60^Table 2 demonstrates phenotype categories along with the total number of phenotypes in that category. The phenotypes fall into categories such as disease or syndrome (N = 788 and %=∼79), drug (N = 142, %=∼14), biomarker (N = 38 and %=∼3.8), lifestyle risk factors (N = 31 and %=3.09), and surgical procedure (N = 1 and %=0.09). The ICD-10 phenotypes illustrate the breadth of the library in terms of diseases: there are 433 phenotypes that include ICD-10 codes, covering all 23 ICD-10 chapters.

**Figure 1.**
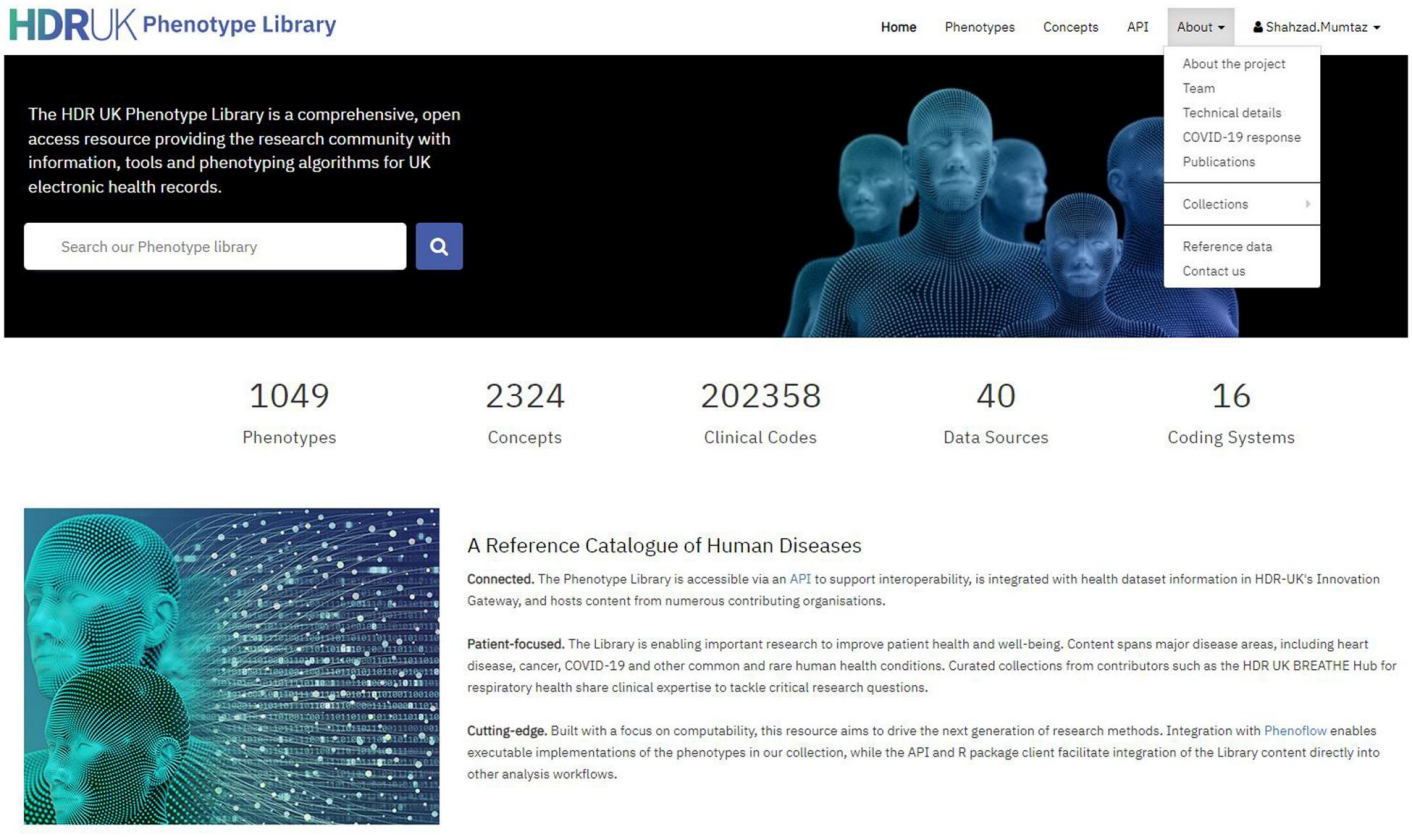
A screenshot of the home page of the HDR UK Phenotype Library.

A screenshot of the Library home page is presented in Figure 1. A description of the phenotype library web pages is provided in Table 1. A sample phenotype page printable version is in the Supplementary material S2.

### Case studies

The Library can support use cases such as using existing phenotype definitions in analysis pipelines and integrating them within decision support systems for clinical guideline development^61–63^ and publishing novel curated phenotypes to be used in other studies.^64–67^ The following examples illustrate current uses of the Library:

#### Intervene

The Intervene project has used the Phenotype Library to assess variability of 40 disease endpoints across international prospective biobank cohorts with the aim of applying and validating Polygenic Scores across several diseases and populations.^61^ The cohorts use different terminologies to describe their data, and endpoints defined within each cohort vary by cohort and coding system. The library has provided community phenotype definitions that have been used in the design of the Intervene endpoint harmonization strategy for current and future endpoints.

#### BREATHE

The BREATHE Health Data Research Hub is a collaboration focused on using data to improve respiratory health. BREATHE researchers, with a goal of “enabling the use of respiratory health data in cutting-edge research and innovation,”^64^ are creating phenotypes of respiratory diseases such as asthma through detailed/fine-grained review of numerous previously published definitions. The phenotype library has enabled sharing these phenotypes, including visual comparison of different published code lists.

#### Adolescent mental health data platform

The library has also been employed by the Adolescent Data Platform for Mental Health (ADP) and DATAMIND (UK’s Hub for Mental Health) researchers to publish their mental health phenotypes.^66^ ADP^65^ and DATAMIND^67^ are also adapting their existing data preparation methods to be driven by these phenotype definitions, demonstrating the value of the library to serve as not just a static documentation resource but a tool integral to research workflows.

#### Multimorbidity tool

Another use case has been in the development of an interactive informatics tool developed for clinical guideline development groups, such as the National Institute for Health and Care Excellence (NICE) and the Scottish Intercollegiate Guidelines Network (SIGN), to compare the properties of trial eligible and ineligible populations.^63^ The tool uses phenotype definitions from the library to define diseases and comorbidities in the analysis pipeline. In its current form, the tool supports 128 conditions as index conditions and 161 conditions as comorbidities to extract meaningful insights from the UK contemporary primary care data linked with hospitalization and mortality. The proof-of-concept tool and was evaluated by the NICE/SIGN users who were very supportive of the use of the tool as an in integral part of the guideline development recommendation process. For example, as part of the tool requirements gathering exercise, a bespoke analysis was conducted for gout guideline development group which was published as appendix to the guideline.^68^

#### British Heart Foundation Data Science Centre

The Phenotype Library has played a leading role in organizing the phenotyping algorithms developed by the British Heart Foundation Data Science Centre (BHF DSC). Created at the start of the COVID-19 pandemic, the BHF DSC enables access to national structured EHR from primary care, hospitalizations, and disease registries from 56 million participants with the focus of providing real-time insights to key policy makers. As part of research carried out in the BHF DSC to inform public health policy, phenotyping algorithms for key outcomes of interest (such as deep vein thrombosis, cardiac arrhythmias, and other cardiometabolic diseases) have been curated on the Phenotype Library and cross linked in publications and technical reports produced.

#### Multimorbidity study

The phenotypes were used in a study to compare the age, sex, and socioeconomic differences in multimorbidity measured in 4 ways within UK primary care data.^62^

## Discussion

### Impact

We set out to build something that would improve the quality and efficiency of health data analysis by facilitating the creation, sharing, and use of phenotypes. The realization of this benefit comes via a combination of the features implemented and the user base reached with the tool.

### Users

As demonstrated by the volume and breadth of content, as well as the highlighted use cases, the Library is actively supporting a wide variety of users and research activities. We believe this success results from the combination of user-focused design and our philosophy of making it an open resource. Analysis of the users visiting the Library demonstrates that over the last twelve months, 3.3K new users visited the Library—predominantly from the UK and then USA, China, Brazil, Spain, India, and Malaysia.

Some good phenotype resources exist for specific projects or settings,^21–27^^,^^29–31^^,^^34^^,^^35^ but the Phenotype Library is the first tool to manage structured phenotype definitions while being open to any user of health data. Anyone can apply for a user account to contribute content, and the phenotype definition has been designed to be as general-purpose as possible, avoiding assumptions that relate to a particular setting.

Key to making this an open resource is our minimal gatekeeping: any phenotype that meets minimum documentation standards is accepted; multiple definitions of the same concept, such as a disease diagnosis, are welcomed. This approach is critical to meeting requirements for transparency and reproducibility through full documentation of analysis methods. Many of the existing resources focus on describing a single, gold standard definition for any concept of interest, such as a disease diagnosis.^19,25^^,^^29–31^ This is important work, but a tool that only enables sharing of approved gold standards cannot be a general solution for reproducibility. The latter requires data users to share their methods at the point of publication—even if it has not been signed off by an authoritative expert group, and even if they did not use the optimal approach. We believe that, rather than hurting quality, more and earlier sharing of work will enable quality improvement in the community over time. This approach does raise the question of how to differentiate between similar phenotypes and select the one that is most appropriate for a given use case. The current version of the Library enables researchers to add their phenotypes which have associated peer reviewed publications such as journal publications, scientific conferences, policy white papers, etc. It does not explicitly enable comparing existing phenotypes; however, we plan to implement a mechanism in the near future to compare phenotypes and develop novel (or improved) phenotypes. The phenotype metadata and references to external resources such as publications currently provide users with information to understand the purpose and assess the quality of content in the Library; in the future, we plan to add more functionality to capture validation information as well as community and expert feedback.

### Comparison to other existing phenotype libraries

The literature demonstrates that often the comparison of phenotype libraries is based on the meta-data and search functionality.^34^^,^^36^ We believe that comparison can be made based on wider criteria as defined in^32^ for the next-generation phenotype libraries (see Table 3).

### Features

As discussed, the feature set of the Library was guided by the principles articulated in our prior work on desiderata for phenotype libraries. Two key groups of features are highlighted here (with a full table in the Supplemental material):

The library is built around structured, comprehensive phenotype metadata. The structure imposed by standardized metadata that is specific to phenotypes has important benefits over using more general-purpose tools. It ensures completeness by covering important points that should be documented, rather than leaving each phenotype creator to start from scratch in deciding what to document; and the standardization imposed facilitates sharing and reuse. If phenotypes share a common structure, it is easier to compare definitions, write general purpose code that can be applied to multiple definitions, swap one phenotype for another in analysis and see the impact, etc.

Furthermore, it was built from the ground up with a focus on programmatic access and computability. Phenotype definitions are one component of the research workflow, and for this tool to be successful, it needs to integrate into a broader technology ecosystem that supports health data analysis. Therefore, having all functionality available via API was a first principle of our work. Existing integrations with the HDR UK Gateway and Phenoflow, as well as the client R package, are demonstrations of interoperability enabled by the API. Furthermore, supporting computability of the phenotypes themselves is an important driver of the benefit of this work. Computable phenotypes move the Library beyond being a documentation resource and toward something that can be integrated directly into data analysis workflows. The 348 phenotypes with linked Phenoflow implementations are one example of the Library’s support for computable definitions, while the API will enable other computability paradigms in the future.

### Plans

We believe this library represents a significant advancement in ability of health data users to manage and share phenotypes. However, this is only a first step, and development is ongoing. Some key areas we have identified for improvement are as follows:

- Storing richer validation information, enabling the community to give feedback, and capturing community reputation.
- Allowing the creation and editing of phenotypes via graphical user interface.
- Supporting more types of content, such as Natural Language Processing or Machine Learning based phenotypes
- Fully computable definitions of phenotypes that include Boolean logic, temporal requirements, and other functionality.
- Engaging health data users to encourage contributions and greater use of the Library.

More details regarding current features and plans for future development are provided in the Supplementary material S3.

## Conclusion

The Phenotype Library is a next-generation platform that meets a clear need of the health data analysis community. Its open access model and features make it a significant new tool for researchers and other data users, supporting repeatability and more efficient work with health data. It is already being used extensively by a broad group of researchers, and we hope that this is just the beginning of its adoption and benefit in the field, driving broader goals of improving population health and wellbeing through gaining insight from health data sources.

## Supplementary Material

ooae049_Supplementary_Data

## Data Availability

All the phenotype definitions are accessible through the HDR UK Phenotype Library and code of the library is accessible through the GitHub.
