# Supplementary material for: Creating a next-generation phenotype library: the health data research UK Phenotype Library
Source: JAMIA Open. 2024 Jun 17;7(2):ooae049. doi: 10.1093/jamiaopen/ooae049 (PMC11182945; doi:10.1093/jamiaopen/ooae049)
Supplement: ooae049_Supplementary_Data [file ooae049_supplementary_data.zip › SupplementaryMaterial_S3.docx]

#### Supplementary Material – S3

Table S3: Current features and plans for future development.

| **Sr. No.** | **Principle** | **Current Features** | **Future Plans** |
| --- | --- | --- | --- |
| 1 | Support modelling languages | Phenotype definitions are structured and rule-based (for code lists); modelling of computable phenotypes as workflows supported via PhenoFLOW | Support modelling of phenotype logic via and integrate with capabilities of PhenoFLOW |
| 2 | Support NLP-based and ML- based definitions | Phenotype model is suitable for text-only descriptions of phenotypes based on natural language processing, machine learning, and other methods | Differentiate between types of phenotypes and capture more domain-specific metadata |
| 3 | Support multi-dimensional descriptions | Ability to describe a high-level phenotype, as well as multiple versions thereof that are specific to individual settings, with their own documentation; as well as to provide an implementation via PhenoFLOW | More structured, differentiated descriptions |
| 4 | Support versioning and data provenance | Version history stored and viewable to users; specific versions referenceable and associated with the user who made changes | Support fuller provenance information |
| 5 | Support modular relationships between phenotypes | A phenotype or a portion thereof can be used within another phenotype | Methods of describing relationships between phenotypes |
| 6 | Communicate implementation information | Enabled via link to PhenoFLOW; further, code lists are stored in a structured manner and accessible via API calls for phenotypes based on clinical codes | Integration of PhenoFLOW and thus tighter connection between abstract definitions and implementations |
| 7 | Support tooling that provides multiple programming language implementations | Standard REST API allows interaction with numerous languages and environments; PhenoFLOW supports multiple languages | Further support to document different types of implementations |
| 8 | Support tooling that provides connectivity with multiple data standards | The Library is data standard agnostic and allows describing phenotypes that can be implemented against any standard | Explicit support for key standards such as the OMOP CDM |
| 9 | Support a defined validation process | Validation information can be captured in the current phenotype structure, but is not an explicit category due to the lack of standardised validation information in the initial phenotype content | Define validation process and standards that can capture the reality of the state of the art while encouraging more rigorous validation |
| 10 | Enable feedback | To be implemented | Explore features such as commenting, voting, etc |
| 11 | Expose a standard API | Standard API covering all functionality | Continue to develop further |
| 12 | Advanced search capabilities | Search based on description, title, author, tags, and contributing organization | Search based on clinical code, further categorizations |
| 13 | Comprehensive metadata | Standard structured phenotype metadata fields supported by flexible tagging system | Support structured data for different phenotype modalities, as well as hierarchical categorization |
