# Supplementary material for: Creating a next-generation phenotype library: the health data research UK Phenotype Library
Source: JAMIA Open. 2024 Jun 17;7(2):ooae049. doi: 10.1093/jamiaopen/ooae049 (PMC11182945; doi:10.1093/jamiaopen/ooae049)
Supplement: ooae049_Supplementary_Data [file ooae049_supplementary_data.zip › SupplementaryMaterial_S1.docx]

#### Supplementary Material S1

Table S1.1: Description of phenotype data structure elements.

| **Field** | **Type** | **Description** |
| --- | --- | --- |
| **Id** | Varchar | e.g. PH123 |
| **Name** | Varchar | Phenotype name |
| **UUID** | Varchrar | Unique ID of original CALIBER portal phenotypes (discontinued in new phenotypes) |
| **Author** | Varchar | List of authors |
| **Type** | Varchar | Phenotype type {biomarker, disease or syndrome, drug, lifestyle risk factor, musculoskeletal, surgical procedure} |
| **Sex** | Varchar | Male/female |
| **valid_event_data_range** | Varchar | From-to date range |
| **validation_performed** | Boolean | Is validated? |
| **Validation** | Longtext | Validation description (accepts markdown) |
| **agreement_date** | Date | Agreement date |
| **Definition** | Longtext | Phenotype definition (accepts markdown) |
| **concept_informations** | Json | List of concept versions containing the relevant code lists from different coding systems |
| **Implementation** | Longtext | Phenotype implementation (accepts markdown) |
| **Tags** | List | List of tags included |
| **Collections** | List | List of collections included |
| **Publications** | List | List of papers/publications **Phenotype Data Structure** |
| **Phenoflowid** | Int | ID to link to PhenoFlow |
| **clinical_terminologies** | List | List of coding systems included |
| **data_sources** | List | List of data sources included |
| **created_by** | User | Creator user |
| **updated_by** | User | Last modifier user |
| **Deleted** | Boolean | Is marked as deleted? |
| **deleted_by** | User | User wo deleted this |
| **Owner** | User | Phenotype owner (controls sharing/permission and publishing) |
| **owner_access** | Int {1= None, 2= View, 3= Edit} | Owner always has Edit permission |
| **Group** | Int | Group ID allowed to have access |
| **group_access** | Int {1= None, 2= View, 3= Edit} | Group access |
| **all_other_users_access** | Int {1= None, 2= View} | Access permission for any other user with a login |
| **History** | HistoricalRecords | Version control of the phenotype |


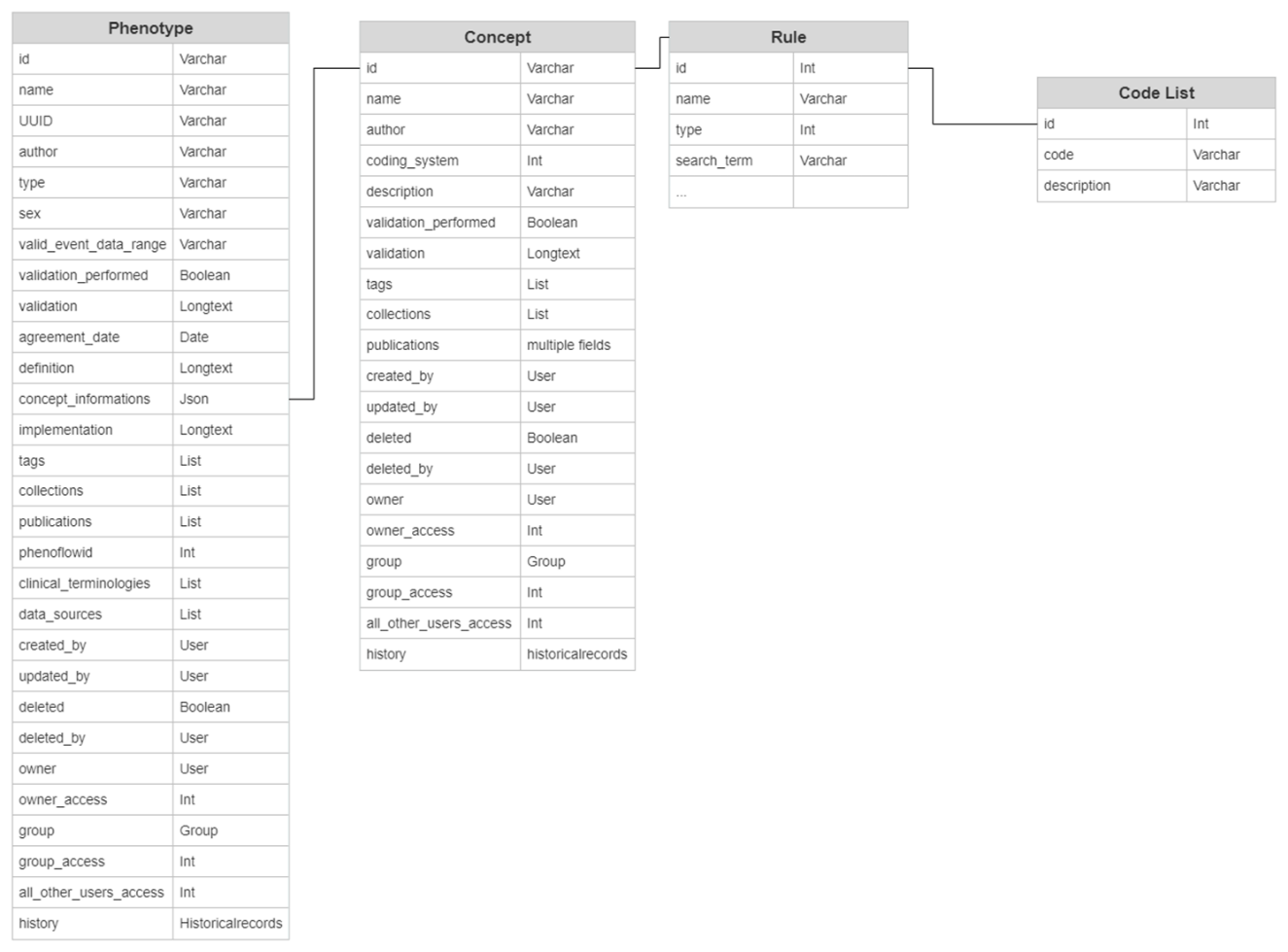


Figure S1.1: Entity-relationship model of phenotypes, concepts and rules (code lists).
