# Supplementary material for: Creating a next-generation phenotype library: the health data research UK Phenotype Library
Source: JAMIA Open. 2024 Jun 17;7(2):ooae049. doi: 10.1093/jamiaopen/ooae049 (PMC11182945; doi:10.1093/jamiaopen/ooae049)
Supplement: ooae049_Supplementary_Data [file ooae049_supplementary_data.zip › SupplementaryMaterial_S2.pdf]

**Supplementary Material - S2**

Phenotypes > **Heart failure**

# Heart failure

*Kuan V, Denaxas S, Gonzalez-Izquierdo A, Direk K, Bhatti O, Husain S, Sutaria S, Hingorani M, Nitsch D, Parisinos C, Lumbers T, Mathur R, Sofat R, Casas JP, Wong I, Hemingway H, Hingorani A*

ID
PH182

Version ID
364

Type
***Disease or Syndrome***

Data Sources
CPRD GOLD , HES Admitted Patient Care data for CPRD GOLD

Valid event data range
01/01/1999 - 01/07/2016

Sex
♀ **Female** ♂ **Male**

Agreement Date
2019-05-20

Coding system
**Read codes v2** **ICD10 codes** **Med codes**

Collections
CALIBER Phenotype Library

This site uses cookies: Learn more





## Definition

At the specified date, a patient is defined as having had ' **Heart failure**' IF they meet the criteria for any of the following on or before the specified date. The earliest date on which the individual meets any of the following criteria on or before the specified date is defined as the first event date:

Primary care

1. ' **Heart failure**' diagnosis or history of diagnosis during a consultation

OR Secondary care (ICD10)

1. ALL diagnoses of ' **Heart failure**' or history of diagnosis during a hospitalization

## Implementation

**PhenoFlow Implementation:**

https://kclhi.org/phenoflow/phenotype/download/88

## Publications

- Kuan V., Denaxas S., Gonzalez-Izquierdo A. et al. A chronological map of 308 physical and mental health conditions from 4 million individuals in the National Health Service. The Lancet Digital Health - DOI 10.1016/S2589-7500(19)30012-3

## Clinical Code List

Expand All / Collapse All

This site uses cookies: Learn more

Rows: 77

| Code | Description | Disease | Medcode | Category |
|---|---|---|---|---|
| 14A6.00 | H/O: **heart failure** | Heart failure | 15058.0 | History of Heart failure |
| 14AM.00 | H/O: **Heart failure** in last year | Heart failure | 46912.0 | History of Heart failure |
| 1O1..00 | **Heart failure** confirmed | Heart failure | 9913.0 | Diagnosis of Heart failure |
| 388D.00 | New York **Heart** Assoc classification **heart failure** symptoms | Heart failure | 46672.0 | Diagnosis of Heart failure |
| 661M500 | **Heart failure** self-management plan agreed | Heart failure | 106198.0 | History of Heart failure |
| 662f.00 | New York **Heart** Association classification - class I | Heart failure | 18853.0 | Diagnosis of Heart failure |
| 662g.00 | New York **Heart** Association classification - class II | Heart failure | 13189.0 | Diagnosis of Heart failure |
| 662h.00 | New York **Heart** Association classification - class III | Heart failure | 19066.0 | Diagnosis of Heart failure |
| 662i.00 | New York **Heart** Association classification - class IV | Heart failure | 51214.0 | Diagnosis of Heart failure |
| 662p.00 | **Heart failure** 6 month review | Heart failure | 83502.0 | History of Heart failure |
| 662T.00 | Congestive **heart failure** monitoring | Heart failure | 12366.0 | History of Heart failure |
| 662W.00 | **Heart failure** annual review | Heart failure | 30779.0 | History of Heart failure |
| 679W100 | Education about deteriorating **heart failure** | Heart failure | 105002.0 | History of Heart failure |
| 679X.00 | **Heart failure** education | Heart failure | 95835.0 | History of Heart failure |
| 8B29.00 | Cardiac **failure** therapy | Heart failure | 24503.0 | Diagnosis of Heart failure |
| 8CeC.00 | Preferred place of care for next exacerbation **heart failure** | Heart failure | 105542.0 | History of Heart failure |

This site uses cookies: Learn more





| Code | Description | Disease | Medcode | Category |
|---|---|---|---|---|
| 8CMK.00 | Has **heart failure** management plan | Heart failure | 103732.0 | History of Heart failure |
| 8CMW800 | **Heart failure** clinical pathway | Heart failure | 106008.0 | History of Heart failure |
| 8H2S.00 | Admit **heart failure** emergency | Heart failure | 32898.0 | History of Heart failure |
| 8HBE.00 | **Heart failure** follow-up | Heart failure | 17851.0 | History of Heart failure |
| 8HHz.00 | Referral to **heart failure** exercise programme | Heart failure | 70619.0 | History of Heart failure |
| 8Hk0.00 | Referred to **heart failure** education group | Heart failure | 71235.0 | History of Heart failure |
| 9h1..00 | Exception reporting: LVD quality indicators | Heart failure | 34213.0 | History of Heart failure |
| 9h11.00 | Excepted from LVD quality indicators: Patient unsuitable | Heart failure | 11613.0 | History of Heart failure |
| 9h12.00 | Excepted from LVD quality indicators: Informed dissent | Heart failure | 28649.0 | History of Heart failure |
| 9hH..00 | Exception reporting: **heart failure** quality indicators | Heart failure | 90935.0 | History of Heart failure |
| 9hH0.00 | Excepted **heart failure** quality indicators: Patient unsuitabl | Heart failure | 30749.0 | History of Heart failure |
| 9hH1.00 | Excepted **heart failure** quality indicators: Informed dissent | Heart failure | 64062.0 | History of Heart failure |
| 9N2p.00 | Seen by community **heart failure** nurse | Heart failure | 19002.0 | History of Heart failure |
| 9N6T.00 | Referred by **heart failure** nurse specialist | Heart failure | 69062.0 | History of Heart failure |
| 9On..00 | Left ventricular dysfunction monitoring administration | Heart failure | 18793.0 | History of Heart failure |
| 9On0.00 | Left ventricular dysfunction monitoring first letter | Heart failure | 60710.0 | History of Heart failure |
| 9On1.00 | Left ventricular dysfunction monitoring second letter | Heart failure | 60721.0 | History of Heart failure |

This site uses cookies: Learn more

| Code | Description | Disease | Medcode | Category |
|---|---|---|---|---|
| 9On3.00 | Left ventricular dysfunction monitoring verbal invite | Heart failure | 92305.0 | History of Heart failure |
| 9On4.00 | Left ventricular dysfunction monitoring telephone invite | Heart failure | 96484.0 | History of Heart failure |
| 9Or..00 | **Heart failure** monitoring administration | Heart failure | 32911.0 | History of Heart failure |
| 9Or0.00 | **Heart failure** review completed | Heart failure | 19380.0 | History of Heart failure |
| 9Or1.00 | **Heart failure** monitoring telephone invite | Heart failure | 90193.0 | History of Heart failure |
| 9Or2.00 | **Heart failure** monitoring verbal invite | Heart failure | 90192.0 | History of Heart failure |
| 9Or3.00 | **Heart failure** monitoring first letter | Heart failure | 72965.0 | History of Heart failure |
| 9Or4.00 | **Heart failure** monitoring second letter | Heart failure | 72386.0 | History of Heart failure |
| 9Or5.00 | **Heart failure** monitoring third letter | Heart failure | 89650.0 | History of Heart failure |
| G1yz100 | Rheumatic left ventricular **failure** | Heart failure | 22262.0 | Diagnosis of Heart failure |
| G210100 | Malignant hypertensive **heart** disease with CCF | Heart failure | 72668.0 | Diagnosis of Heart failure |
| G211100 | Benign hypertensive **heart** disease with CCF | Heart failure | 52127.0 | Diagnosis of Heart failure |
| G21z100 | Hypertensive **heart** disease NOS with CCF | Heart failure | 62718.0 | Diagnosis of Heart failure |
| G232.00 | Hypertensive **heart**&renal dis wth (congestive) **heart failure** | Heart failure | 21837.0 | Diagnosis of Heart failure |
| G234.00 | Hyperten **heart**&renal dis+both(congestv)**heart** and renal fail | Heart failure | 57987.0 | Diagnosis of Heart failure |
| G400.00 | Acute cor pulmonale | Heart failure | 8464.0 | Diagnosis of Heart failure |

This site uses cookies: Learn more





| Code | Description | Disease | Medcode | Category |
|---|---|---|---|---|
| G554000 | Congestive cardiomyopathy | Heart failure | 5141.0 | Diagnosis of Heart failure |
| G554011 | Congestive obstructive cardiomyopathy | Heart failure | 68766.0 | Diagnosis of Heart failure |
| G58..00 | **Heart failure** | Heart failure | 2062.0 | Diagnosis of Heart failure |
| G58..11 | Cardiac **failure** | Heart failure | 1223.0 | Diagnosis of Heart failure |
| G580.00 | Congestive **heart failure** | Heart failure | 398.0 | Diagnosis of Heart failure |
| G580.11 | Congestive cardiac **failure** | Heart failure | 2906.0 | Diagnosis of Heart failure |
| G580.12 | Right **heart failure** | Heart failure | 10079.0 | Diagnosis of Heart failure |
| G580.13 | Right ventricular **failure** | Heart failure | 10154.0 | Diagnosis of Heart failure |
| G580.14 | Biventricular **failure** | Heart failure | 9524.0 | Diagnosis of Heart failure |
| G580000 | Acute congestive **heart failure** | Heart failure | 23707.0 | Diagnosis of Heart failure |
| G580100 | Chronic congestive **heart failure** | Heart failure | 32671.0 | Diagnosis of Heart failure |
| G580200 | Decompensated cardiac **failure** | Heart failure | 27884.0 | Diagnosis of Heart failure |
| G580300 | Compensated cardiac **failure** | Heart failure | 11424.0 | Diagnosis of Heart failure |
| G580400 | Congestive **heart failure** due to valvular disease | Heart failure | 94870.0 | Diagnosis of Heart failure |
| G581.00 | Left ventricular **failure** | Heart failure | 884.0 | Diagnosis of Heart failure |
| G581.11 | Asthma - cardiac | Heart failure | 23481.0 | Diagnosis of Heart failure |
| G581.13 | Impaired left ventricular function | Heart failure | 5942.0 | Diagnosis of Heart failure |

This site uses cookies: Learn more

| Code | Description | Disease | Medcode | Category |
|---|---|---|---|---|
| G582.00 | Acute **heart failure** | Heart failure | 27964.0 | Diagnosis of Heart failure |
| G584.00 | Right ventricular **failure** | Heart failure | 104275.0 | Diagnosis of Heart failure |
| G58z.00 | **Heart failure** NOS | Heart failure | 4024.0 | Diagnosis of Heart failure |
| G58z.12 | Cardiac **failure** NOS | Heart failure | 17278.0 | Diagnosis of Heart failure |
| G5yy900 | Left ventricular systolic dysfunction | Heart failure | 8966.0 | Diagnosis of Heart failure |
| G5yyA00 | Left ventricular diastolic dysfunction | Heart failure | 12550.0 | Diagnosis of Heart failure |
| ZRad.00 | New York **Heart** Assoc classification **heart failure** symptoms | Heart failure | 26242.0 | Diagnosis of Heart failure |

Showing 1 to 77 of 77 entries

## C1207/3553 Heart failure - Primary care - Med codes

Rows: 77

| Code | Description | Disease | ReadcodeDescr | Readcode |
|---|---|---|---|---|
| 398.0 | Diagnosis of **Heart failure** | Heart failure | Congestive heart failure | G580.00 |
| 884.0 | Diagnosis of **Heart failure** | Heart failure | Left ventricular failure | G581.00 |
| 1223.0 | Diagnosis of **Heart failure** | Heart failure | Cardiac failure | G58..11 |
| 2062.0 | Diagnosis of **Heart failure** | Heart failure | Heart failure | G58..00 |
| 2906.0 | Diagnosis of **Heart failure** | Heart failure | Congestive cardiac failure | G580.11 |
| [illegible] | [illegible] | [illegible] | [illegible] | [illegible] |

This site uses cookies: Learn more





| Code | Description | Disease | ReadcodeDescr | Readcode |
|---|---|---|---|---|
| 5255.0 | Diagnosis of **Heart failure** | Heart failure | Acute left ventricular failure | G581000 |
| 5695.0 | Diagnosis of **Heart failure** | Heart failure | Chronic cor pulmonale | G41z.11 |
| 5942.0 | Diagnosis of **Heart failure** | Heart failure | Impaired left ventricular function | G581.13 |
| 8464.0 | Diagnosis of **Heart failure** | Heart failure | Acute cor pulmonale | G400.00 |
| 8966.0 | Diagnosis of **Heart failure** | Heart failure | Left ventricular systolic dysfunction | G5yy900 |
| 9524.0 | Diagnosis of **Heart failure** | Heart failure | Biventricular failure | G580.14 |
| 9913.0 | Diagnosis of **Heart failure** | Heart failure | Heart failure confirmed | 1O1..00 |
| 10079.0 | Diagnosis of **Heart failure** | Heart failure | Right heart failure | G580.12 |
| 10154.0 | Diagnosis of **Heart failure** | Heart failure | Right ventricular failure | G580.13 |
| 11424.0 | Diagnosis of **Heart failure** | Heart failure | Compensated cardiac failure | G580300 |
| 11613.0 | History of **Heart failure** | Heart failure | Excepted from LVD quality indicators: Patient unsuitable | 9h11.00 |
| 12366.0 | History of **Heart failure** | Heart failure | Congestive heart failure monitoring | 662T.00 |
| 12550.0 | Diagnosis of **Heart failure** | Heart failure | Left ventricular diastolic dysfunction | G5yyA00 |
| 13189.0 | Diagnosis of **Heart failure** | Heart failure | New York Heart Association classification - class II | 662g.00 |
| 15058.0 | History of **Heart failure** | Heart failure | H/O: heart failure | 14A6.00 |
| 17278.0 | Diagnosis of **Heart failure** | Heart failure | Cardiac failure NOS | G58z.12 |
| 17851.0 | History of **Heart failure** | Heart failure | Heart failure follow-up | 8HBE.00 |

This site uses cookies: Learn more

| Code | Description | Disease | ReadcodeDescr | Readcode |
|---|---|---|---|---|
| 18853.0 | Diagnosis of **Heart failure** | Heart failure | New York Heart Association classification - class I | 662f.00 |
| 19002.0 | History of **Heart failure** | Heart failure | Seen by community heart failure nurse | 9N2p.00 |
| 19066.0 | Diagnosis of **Heart failure** | Heart failure | New York Heart Association classification - class III | 662h.00 |
| 19380.0 | History of **Heart failure** | Heart failure | Heart failure review completed | 9Or0.00 |
| 21837.0 | Diagnosis of **Heart failure** | Heart failure | Hypertensive heart&renal dis wth (congestive) heart failure | G232.00 |
| 22262.0 | Diagnosis of **Heart failure** | Heart failure | Rheumatic left ventricular failure | G1yz100 |
| 23481.0 | Diagnosis of **Heart failure** | Heart failure | Asthma - cardiac | G581.11 |
| 23707.0 | Diagnosis of **Heart failure** | Heart failure | Acute congestive heart failure | G580000 |
| 24503.0 | Diagnosis of **Heart failure** | Heart failure | Cardiac failure therapy | 8B29.00 |
| 26242.0 | Diagnosis of **Heart failure** | Heart failure | New York Heart Assoc classification heart failure symptoms | ZRad.00 |
| 27884.0 | Diagnosis of **Heart failure** | Heart failure | Decompensated cardiac failure | G580200 |
| 27964.0 | Diagnosis of **Heart failure** | Heart failure | Acute heart failure | G582.00 |
| 28649.0 | History of **Heart failure** | Heart failure | Excepted from LVD quality indicators: Informed dissent | 9h12.00 |
| 30749.0 | History of **Heart failure** | Heart failure | Excepted heart failure quality indicators: Patient unsuitabl | 9hH0.00 |
| 30779.0 | History of **Heart failure** | Heart failure | Heart failure annual review | 662W.00 |
| 32671.0 | Diagnosis of **Heart failure** | Heart failure | Chronic congestive heart failure | G580100 |

This site uses cookies: Learn more





| Code | Description | Disease | ReadcodeDescr | Readcode |
|---|---|---|---|---|
| 32911.0 | History of **Heart failure** | Heart failure | Heart failure monitoring administration | 9Or..00 |
| 32945.0 | History of **Heart failure** | Heart failure | Heart failure care plan discussed with patient | 8CL3.00 |
| 34213.0 | History of **Heart failure** | Heart failure | Exception reporting: LVD quality indicators | 9h1..00 |
| 46672.0 | Diagnosis of **Heart failure** | Heart failure | New York Heart Assoc classification heart failure symptoms | 388D.00 |
| 46912.0 | History of **Heart failure** | Heart failure | H/O: Heart failure in last year | 14AM.00 |
| 51214.0 | Diagnosis of **Heart failure** | Heart failure | New York Heart Association classification - class IV | 662i.00 |
| 52127.0 | Diagnosis of **Heart failure** | Heart failure | Benign hypertensive heart disease with CCF | G211100 |
| 57987.0 | Diagnosis of **Heart failure** | Heart failure | Hyperten heart&renal dis+both(congestv)heart and renal fail | G234.00 |
| 60710.0 | History of **Heart failure** | Heart failure | Left ventricular dysfunction monitoring first letter | 9On0.00 |
| 60721.0 | History of **Heart failure** | Heart failure | Left ventricular dysfunction monitoring second letter | 9On1.00 |
| 62718.0 | Diagnosis of **Heart failure** | Heart failure | Hypertensive heart disease NOS with CCF | G21z100 |
| 64062.0 | History of **Heart failure** | Heart failure | Excepted heart failure quality indicators: Informed dissent | 9hH1.00 |
| 68766.0 | Diagnosis of **Heart failure** | Heart failure | Congestive obstructive cardiomyopathy | G554011 |
| 69062.0 | History of **Heart failure** | Heart failure | Referred by heart failure nurse specialist | 9N6T.00 |
| 70619.0 | History of **Heart failure** | Heart failure | Referral to heart failure exercise programme | 8HHz.00 |
| 71235.0 | History of **Heart failure** | Heart failure | Referred to heart failure education group | 8Hk0.00 |

This site uses cookies: Learn more

| Code | Description | Disease | ReadcodeDescr | Readcode |
|---|---|---|---|---|
| 72386.0 | History of **Heart failure** | Heart failure | Heart failure monitoring second letter | 9Or4.00 |
| 72668.0 | Diagnosis of **Heart failure** | Heart failure | Malignant hypertensive heart disease with CCF | G210100 |
| 72965.0 | History of **Heart failure** | Heart failure | Heart failure monitoring first letter | 9Or3.00 |
| 83502.0 | History of **Heart failure** | Heart failure | Heart failure 6 month review | 662p.00 |
| 89650.0 | History of **Heart failure** | Heart failure | Heart failure monitoring third letter | 9Or5.00 |
| 90192.0 | History of **Heart failure** | Heart failure | Heart failure monitoring verbal invite | 9Or2.00 |
| 90193.0 | History of **Heart failure** | Heart failure | Heart failure monitoring telephone invite | 9Or1.00 |
| 90935.0 | History of **Heart failure** | Heart failure | Exception reporting: heart failure quality indicators | 9hH..00 |
| 92305.0 | History of **Heart failure** | Heart failure | Left ventricular dysfunction monitoring verbal invite | 9On3.00 |
| 94870.0 | Diagnosis of **Heart failure** | Heart failure | Congestive heart failure due to valvular disease | G580400 |
| 95835.0 | History of **Heart failure** | Heart failure | Heart failure education | 679X.00 |
| 96484.0 | History of **Heart failure** | Heart failure | Left ventricular dysfunction monitoring telephone invite | 9On4.00 |
| 103732.0 | History of **Heart failure** | Heart failure | Has heart failure management plan | 8CMK.00 |
| 104275.0 | Diagnosis of **Heart failure** | Heart failure | Right ventricular failure | G584.00 |
| 105002.0 | History of **Heart failure** | Heart failure | Education about deteriorating heart failure | 679W100 |
| 105542.0 | History of **Heart failure** | Heart failure | Preferred place of care for next exacerbation heart failure | 8CeC.00 |
| 106008.0 | History of **Heart failure** | Heart failure | Heart failure clinical pathway | 8CMW800 |

This site uses cookies: Learn more





Showing 1 to 77 of 77 entries

**━ C1208/3555 Heart failure - Secondary care - Diagnoses - ICD10 codes**

Rows: 4

| Code | Description | Disease | Category |
|---|---|---|---|
| I11.0 | Hypertensive **heart** disease with (congestive) **heart failure** | Heart failure | Diagnosis of Heart failure |
| I13.0 | Hypertensive **heart** and renal disease with (congestive) **heart failure** | Heart failure | Diagnosis of Heart failure |
| I13.2 | Hypertensive **heart** and renal disease with both (congestive) **heart failure** and renal **failure** | Heart failure | Diagnosis of Heart failure |
| I50 | **Heart failure** | Heart failure | Diagnosis of Heart failure |

Showing 1 to 4 of 4 entries

# API

To Export Phenotype Details:

| Format | API |
|---|---|
| XML | http://phenotypes.healthdatagateway.org/api/v1/public/phenotypes/PH182/version/364/detail/?format=xml |
| JSON | http://phenotypes.healthdatagateway.org/api/v1/public/phenotypes/PH182/version/364/detail/?format=json |

This site uses cookies: Learn more

| | |
|---|---|
| R Package | `# Download here`<br>`library(ConceptLibraryClient)`<br><br>`# Connect to API`<br>`client = connect_to_API(public=TRUE)`<br><br>`# Get details of phenotype`<br>`details = get_phenotype_detail_by_version('PH182', '364', api_client=client)` |

To Export Phenotype Code List:

| Format | API |
|---|---|
| XML | http://phenotypes.healthdatagateway.org/api/v1/public/phenotypes/PH182/version/364/export/codes/?format=xml |
| JSON | http://phenotypes.healthdatagateway.org/api/v1/public/phenotypes/PH182/version/364/export/codes/?format=json |
| CSV | http://phenotypes.healthdatagateway.org/phenotypes/PH182/version/364/export/codes/ |
| R Package | `# Download here`<br>`library(ConceptLibraryClient)`<br><br>`# Connect to API`<br>`client = connect_to_API(public=TRUE)`<br><br>`# Get codelists of phenotype`<br>`codelists = get_phenotype_code_list('PH182', '364', api_client=client)` |

## Version History

| Version ID | Name | Owner | Publish date |
|---|---|---|---|

This site uses cookies: Learn more





This site uses cookies: Learn more
